# Virtual Reality for Perioperative Care in Cardiac Surgery: A Systematic Review and Meta-Analysis

**DOI:** 10.7759/cureus.97819

**Published:** 2025-11-25

**Authors:** Ali Alseneid, Harvey Xiang, Alhassen Alseneid, Marwan Ibrahim, Shivam Bhindi

**Affiliations:** 1 Internal Medicine, William Harvey Hospital, Ashford, GBR; 2 Internal Medicine, Princess Royal Hospital, Haywards Heath, GBR; 3 General Surgery, Milton Keynes University Hospital, Milton Keynes, GBR; 4 Internal Medicine, King's College London GKT School of Medical Education, London, GBR

**Keywords:** adult cardiac surgery, immersive technologies, immersive virtual reality, perioperative anxiety, perioperative outcomes, perioperative pain management, systematic review and meta-analysis

## Abstract

Cardiac surgery imposes substantial physiological and psychological stress, with preoperative anxiety and postoperative pain persisting despite advances in perioperative care. Elevated anxiety has been associated with longer recovery and poorer outcomes, emphasizing the need for adjunctive, nonpharmacological interventions. Virtual reality (VR), an immersive digital technology that modulates sensory perception and emotional responses, has shown benefit in other surgical fields but has not been systematically evaluated in cardiac surgery. This systematic review and meta-analysis provide the first comprehensive synthesis of randomized controlled trials (RCTs) assessing VR in adult cardiac surgery. Following the Preferred Reporting Items for Systematic reviews and Meta-Analyses (PRISMA) 2020 guidelines and International Prospective Register of Systematic Reviews (PROSPERO) registration, PubMed, Embase, and Cochrane Library were searched from inception to October 2025. Primary outcomes were anxiety, pain, and satisfaction; secondary outcomes included physiological responses, opioid use, and adverse events. Risk of bias was assessed using the RoB 2 tool, and evidence certainty was evaluated with the Grading of Recommendations Assessment, Development and Evaluation (GRADE) approach. The data were synthesized using a random-effects meta-analysis. Nine RCTs encompassing 888 participants were included. Meta-analysis of seven trials showed a nonsignificant trend toward lower preoperative anxiety with VR versus control (standardized mean difference (SMD) = -0.23; 95% CI -0.50 to 0.04, low heterogeneity (I² = 37%)). Four studies assessing patient satisfaction also showed a nonsignificant favor of VR (SMD = 0.36; 95% CI -0.65 to 1.38; I² = 92%). While two studies found no consistent analgesic benefit, physiological data suggested VR may attenuate autonomic arousal, lowering heart rate and blood pressure in select settings, though findings remained heterogeneous. Across all trials, VR was well tolerated, with only mild, transient adverse events reported. VR represents a feasible and safe perioperative adjunct in cardiac surgery, demonstrating favorable, nonsignificant trends in anxiety reduction and patient satisfaction. Further large-scale, standardized RCTs are warranted to confirm its physiological and clinical utility.

## Introduction and background

Cardiac surgery, including coronary artery bypass grafting (CABG) and valve operations, continues to be performed at high volumes across both Europe and the United States [1,2]. National registry analyses consistently highlight its central role in the management of advanced cardiovascular disease, with tens of thousands of procedures undertaken annually and outcomes that remain a major focus of quality improvement initiatives [1,2]. Despite advances in perioperative care, cardiac surgery continues to be associated with significant psychological and physiological stress, including impaired quality of life and heightened levels of anxiety, particularly in the perioperative period [3]. An observational study by AbuRuz et al. demonstrated that higher preoperative anxiety was significantly associated with prolonged hospital length of stay following CABG [4]. Similarly, Tasbihgou et al. found that psychological and behavioral factors, including preoperative anxiety, were linked to an increased risk of postoperative neurocognitive disorder and delayed recovery in this population [5]. Furthermore, poorly controlled perioperative pain and distress can diminish patient satisfaction and impede functional recovery [6]. Collectively, these outcomes highlight the importance of effective perioperative interventions aimed not only at improving surgical success but also at enhancing patient experience and subsequent postoperative prognosis.

Virtual reality (VR) has emerged as an encouraging adjunctive technology in healthcare, offering immersive environments that can modulate attention, reduce pain perception, and alleviate anxiety during medical procedures [7]. The evidence across surgical specialties, including orthopedics, otolaryngology, and minimally invasive interventions, has demonstrated that VR can enhance patient comfort and perioperative experiences [8-10]. Within cardiology, VR has recently been evaluated in interventional settings such as percutaneous coronary intervention (PCI) and catheter ablation. A recent systematic review and meta-analysis of RCTs found that VR significantly reduced peri-procedural anxiety in patients undergoing cardiac procedures, although evidence for its effectiveness in pain reduction was inconclusive [11]. Despite these encouraging findings, the application of VR specifically in cardiac surgery, a setting associated with uniquely high levels of psychological distress and physiological stressors, has not yet been systematically synthesized.

As immersive technologies become increasingly integrated into perioperative practice, their potential to enhance patient-centered outcomes in cardiac surgery warrants rigorous evaluation. This review seeks to provide the first comprehensive synthesis of randomized controlled evidence examining the effectiveness of VR interventions in this setting. Specifically, we assess whether VR improves key perioperative outcomes, including preoperative anxiety, postoperative pain, patient satisfaction, and other important outcomes, compared with standard care or control conditions.

## Review

Methodology

Protocol and Registration

This systematic review and meta-analysis adhered to the Preferred Reporting Items for Systematic reviews and Meta-Analyses (PRISMA) 2020 guidelines. The review protocol was prospectively registered with PROSPERO (registration ID: CRD420251159413). The protocol details are publicly available.

Search Strategy

A comprehensive literature search of PubMed, Embase, and Cochrane CENTRAL was undertaken from database inception to October 2025. The search combined controlled vocabulary and free-text terms related to VR and cardiac surgery, including (“virtual reality” OR “VR” OR “immersive video” OR “3-Dimensional video”) AND (“cardiac surgery” OR “open heart surgery” OR “coronary artery bypass grafting” OR “valve surgery”) AND (“anxiety” OR “pain” OR “patient satisfaction”). The reference lists of all included studies and relevant reviews were also screened to identify additional eligible articles.

Studies were considered eligible if they were RCTs that evaluated the use of a VR intervention in adults undergoing cardiac surgery in the perioperative period. Only trials that included a control group receiving standard care or a non-VR intervention were included. Nonrandomized designs, conference abstracts, case reports, reviews, and studies involving nonsurgical cardiac procedures such as PCI or catheter ablation were excluded. Studies involving pediatric populations or those not available in English were also excluded.

Study Selection and Data Extraction

Two reviewers (AA and HX) independently screened titles and abstracts for eligibility, followed by full-text review. All duplicates were removed using Rayyan software (www.rayyan.ai), and discrepancies were resolved through discussion or consultation with the remaining others. Data extraction was performed independently by two reviewers (AA and HX) using a standardized template, and variables included study design, sample size, patient demographics, type and timing of surgery, VR intervention details, comparator group, and all reported outcomes. Primary outcomes of interest were preoperative anxiety, pain, and patient satisfaction. Secondary outcomes included physiologic measures (heart rate, blood pressure, respiratory rate, and oxygen saturation), opioid use, and adverse events.

Risk of Bias Assessment

Risk of bias was assessed using the Cochrane Risk of Bias 2.0 (RoB 2.0) tool for RCTs [12]. Five domains were evaluated: randomization process, deviations from intended interventions, missing outcome data, measurement of outcomes, and selection of reported results. Each study was rated as low risk, some concerns, or high risk.

Certainty of Evidence Assessment

The overall confidence in the evidence for each outcome was evaluated using the GRADE approach. This process considered key domains, including study limitations, inconsistency of results, indirectness of evidence, imprecision, and potential publication bias.

Statistical Analysis

For continuous outcomes, effect sizes were expressed as standardized mean differences (SMDs) with 95% CIs, using Hedges’ g correction to account for small sample sizes. Meta-analyses were performed using a random-effects model (DerSimonian-Laird method), given the expected clinical and methodological heterogeneity across studies. Statistical heterogeneity was quantified using the I² statistic. A Knapp-Hartung adjustment was applied to improve CI estimation in analyses with fewer than 10 studies. All analyses were performed using the online platform MetaAnalysisOnline.com.

When sufficient studies reported an outcome (≥3 RCTs), pooled estimates were calculated and presented as forest plots. Funnel plots were generated to assess small-study effects and potential publication bias. When meta-analysis was not possible, a narrative synthesis was conducted.

Results

Study Selection and Study Characteristics

**Figure 1:**
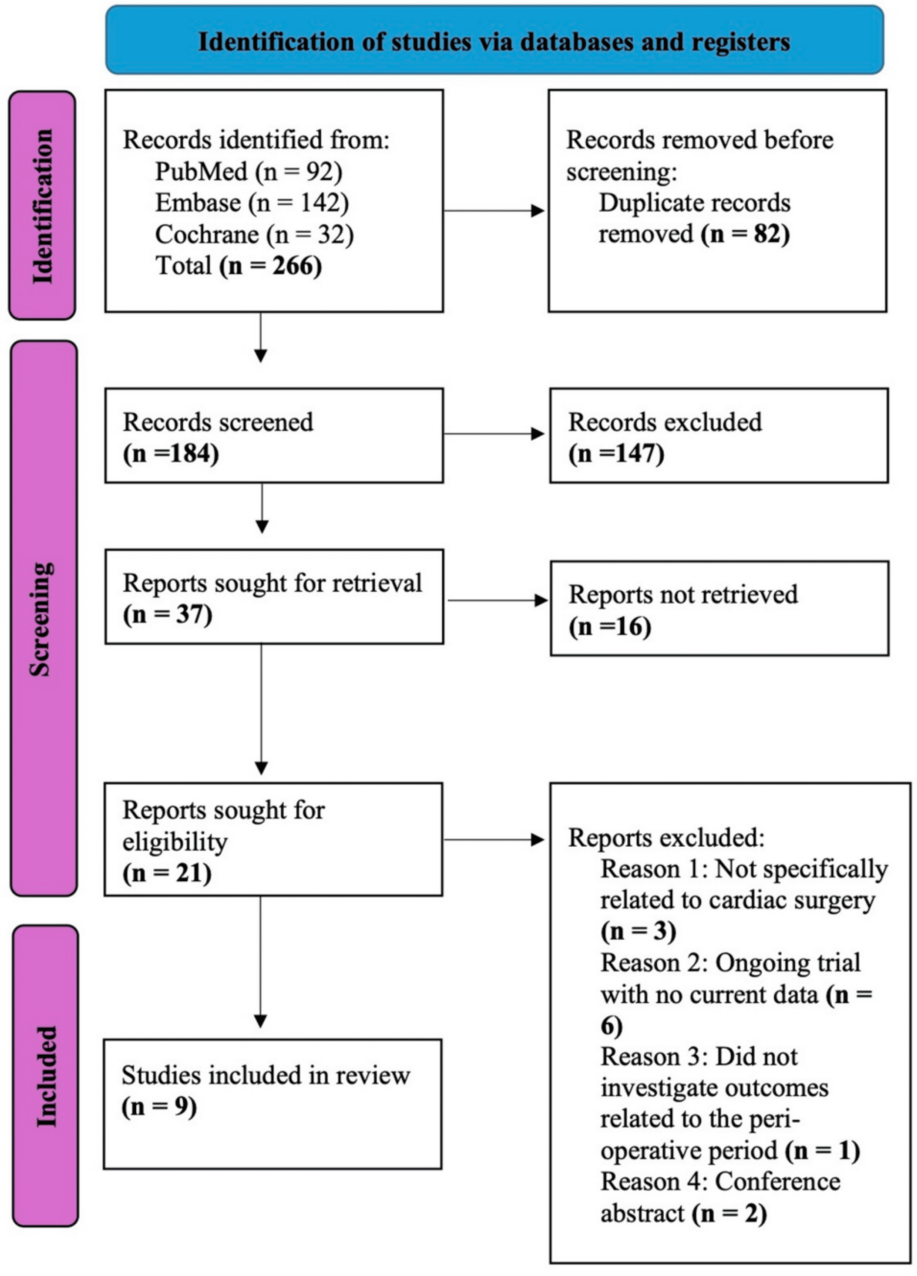
PRISMA flow diagram illustrating the study selection process for inclusion in the systematic review and meta-analysis A total of 266 records were retrieved, with nine studies meeting the inclusion criteria and incorporated into the final analysis. PRISMA, Preferred Reporting Items for Systematic reviews and Meta-Analyses

The initial search of PubMed, Embase, and Cochrane CENTRAL identified 266 records. After removal of duplicates (n = 82), 184 records underwent title and abstract screening, of which 147 were excluded as not meeting the inclusion criteria. A total of 37 full-text reports were assessed for eligibility, with 16 not retrieved. Of the 21 reports reviewed, 12 were excluded, and ultimately, nine RCTs were included in the final synthesis. The study selection process is seen in the PRISMA flow diagram in Figure 1.

The nine studies, published between 2020 and 2025, were conducted across Europe, North America, Asia, and Russia [13-21]. Collectively, these trials enrolled 888 participants, with mean ages ranging from 50 to 67 years and predominantly male cohorts. All studies focused on adult patients undergoing cardiac surgery.

VR interventions varied in design and application but consistently utilized immersive environments during the perioperative period. These included 360° hospital and operating theater tours, interactive 3D cardiac models, nature- and relaxation-based experiences, and cognitive training modules. Most interventions were delivered preoperatively as a single session, while several incorporated multi-session or postoperative components [13-21]. Control groups included standard education, 2D videos, printed materials, or conventional care.

Primary outcomes commonly assessed were preoperative anxiety, postoperative pain, and patient satisfaction, measured using validated instruments such as the State-Trait Anxiety Inventory (STAI), the Amsterdam Preoperative Anxiety and Information Scale (APAIS), and the Visual Analogue Scale (VAS) or Numerical Rating Scale (NRS). Secondary outcomes included physiological parameters (e.g., heart rate, blood pressure, respiratory rate, and oxygen saturation), opioid use, and adverse effects. A detailed summary of study characteristics is presented in Table 1.

**Table 1:** Summary of key features of the studies included in the systematic review and meta-analysis evaluating VR interventions during the perioperative period for patients undergoing cardiac surgery AVR, aortic valve replacement; CABG, coronary artery bypass grafting; MVR, mitral valve replacement; RCT, randomized controlled trial; SAVR, surgical aortic valve repair; TAA, thoracic aortic aneurysm; VR, virtual reality; VRH, virtual reality hypnosis

Study	Design	Participants (M/F)	Population	Procedure	VR type	VR content	Control group intervention
El Mathari et al. [13]	RCT (single-center)	121 (98M/23F)	Adults (CABG/valve surgery via sternotomy)	CABG/valve surgery	360° VR animation	Patient journey + 3D animation	Oral + flyer traditional education
Subramaniam et al. [14]	RCT (single-center)	100 (65M/35F)	Adults (21-80 years) first-time sternotomy	First-time sternotomy (CABG/valve)	Immersive VR (Oculus)	Nature and seasonal scenes	Tablet with the same non-immersive content
Grab et al. [15]	RCT (monocentric)	99 (87M/12F)	Adults ≥18 years scheduled for CABG, SAVR, and TAA	CABG, SAVR, and TAA	Oculus Quest 2	Interactive 3D surgical models	Paper-based education + 3D models
Amiri et al. [16]	RCT (interventional-educational approach)	60 (38M/22F)	Adults (30-70 years) open heart surgery (CABG)	Open heart surgery (CABG)	TSCO VR glasses	360° VR orientation video	Ordinary 2D video
Tarasova et al. [17]	RCT (single-center)	100 (100M/0F)	Adults (45-75 years) elective CABG	CABG	HP Reverb G2 VR headset	Multitask VR driving + cognitive task	Standard postoperative care
Laghlam et al. [18]	Prospective RCT (noninferiority)	200 (134M/46F)	Adults >18 years, chest drain removal post-cardiac surgery	Chest drain removal post-cardiac surgery	Think Reality VRX headset	360° relaxing immersive environments	Kalinox (nitrous oxide/oxygen mixture)
Rousseaux et al. [19]	Prospective RCT, four-arm	100 (76M/24F)	Adults undergoing CABG, AVR, and MVR	CABG, AVR, and MVR	Oncomfort Sedakit VR headset	Passive VR (landscape, sunrise/clouds)	Standard care; comparator arms: hypnosis and VRH
Sadlonova et al. [20]	Three-arm RCT	88 (69M/19F)	Adults (≥18 yrs) elective CABG	CABG	VR (within multimodal package)	Relaxing VR images + music, light therapy, noise reduction	Standard medical care
Hendricks et al. [21]	RCT pilot (single-blind)	20 (18M/2F)	Adults undergoing first-time sternotomy	CABG/valve surgery	Samsung Gear Oculus	Interactive VR game	Tablet-based non-VR game

**Figure 2:**
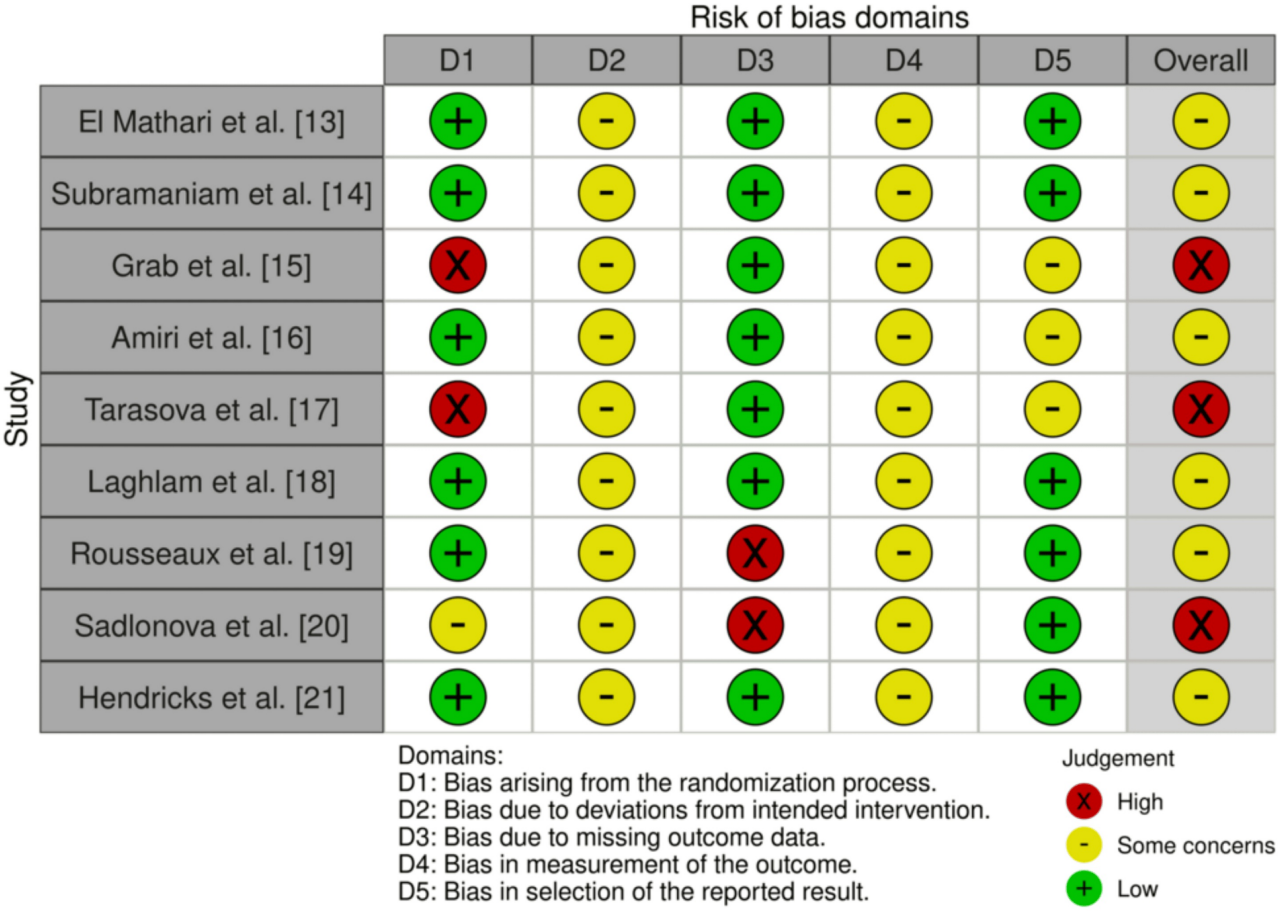
RoB 2 tool assessing risk of bias in RCT studies Summary of risk-of-bias evaluations for the RCTs included in the review, assessed using the Cochrane RoB 2.0 tool. Visualization was generated with the RobVis online platform. RCT, randomized controlled trial

Risk of bias assessment using the RoB 2.0 tool indicated that the majority of trials were judged as having “some concerns,” primarily due to issues related to blinding, deviations from intended interventions, and selective outcome reporting. Three studies were rated at high risk of bias, largely because of limitations in the randomization process and missing outcome data [15,17,20]. The remaining studies were judged to be at low risk in most domains, though concerns regarding the measurement of subjective outcomes (e.g., anxiety and pain scores) and allocation concealment were common. The risk of bias assessment is demonstrated in Figure 2.

Effect of VR on Preoperative Anxiety of Cardiac Surgery Patients

Of the nine included RCTs, seven reported quantitative preoperative anxiety outcomes using validated measures (STAI, VAS, APAIS, or NRS) [13-16,18,19,21]. Two studies were excluded: Tarasova et al., which assessed only neurophysiological markers, and Sadlonova et al., where VR was embedded within a multimodal intervention and anxiety could not be isolated [17,20].

**Figure 3:**
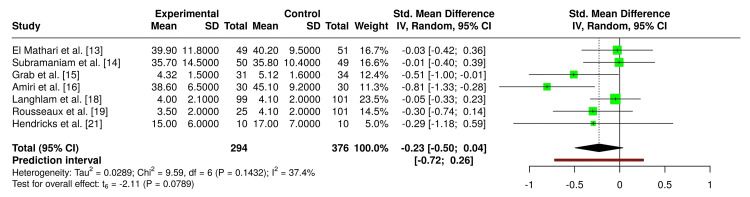
Forest plot comparing VR versus control management for preoperative anxiety scores in cardiac surgery patients (random-effects meta-analysis) Forest plot comparing VR interventions with standard care or control conditions for preoperative anxiety in patients undergoing cardiac surgery. The analysis demonstrated a nonsignificant trend toward reduced anxiety with VR compared to controls, based on a random-effects meta-analysis. VR, virtual reality

**Figure 4:**
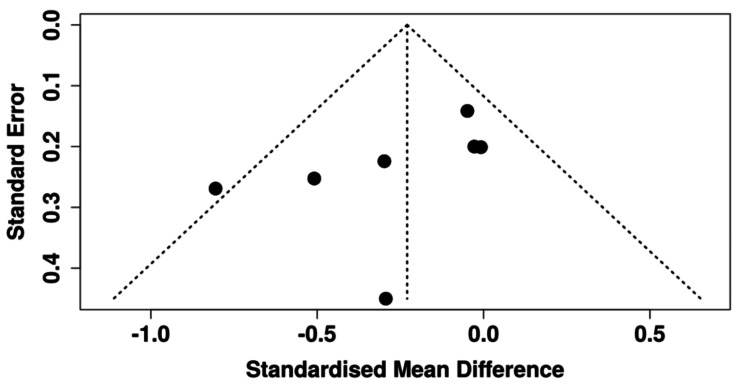
Funnel plot evaluating potential publication bias for preoperative anxiety outcomes in cardiac surgery patients Funnel plot illustrating the distribution of studies assessing preoperative anxiety outcomes. The plot demonstrates a balanced spread of effect sizes around the pooled estimate, indicating minimal evidence of publication bias or small-study effects [13-16,18,19,21].

A random-effects meta-analysis of the seven eligible trials, including 670 participants (294 VR, 376 controls), demonstrated a nonsignificant trend toward reduced anxiety with VR compared to controls (SMD = -0.23, 95% CI -0.50 to 0.04; p = 0.079) [13-16,18,19,21]. Heterogeneity was low (I² = 37.4%), and funnel plot inspection and Egger’s regression test (p = 0.15) did not suggest evidence of publication bias. These results are visualized in the forest plot in Figure 3 and the funnel plot in Figure 4.

Individual trial results were mixed. Amiri et al. and Hendricks et al. demonstrated significant anxiety reduction in the VR group compared to controls, while Subramaniam et al. observed significant within-group reductions in VR but no superiority to tablet-based comparators [14,16,21]. Grab et al. found significant improvement in VAS anxiety but not in STAI, whereas El Mathari et al., Rousseaux et al., and Laghlam et al. reported no significant between-group differences [13,15,18,19].

Effect of VR on Patient Satisfaction in Cardiac Surgery Patients

**Figure 5:**
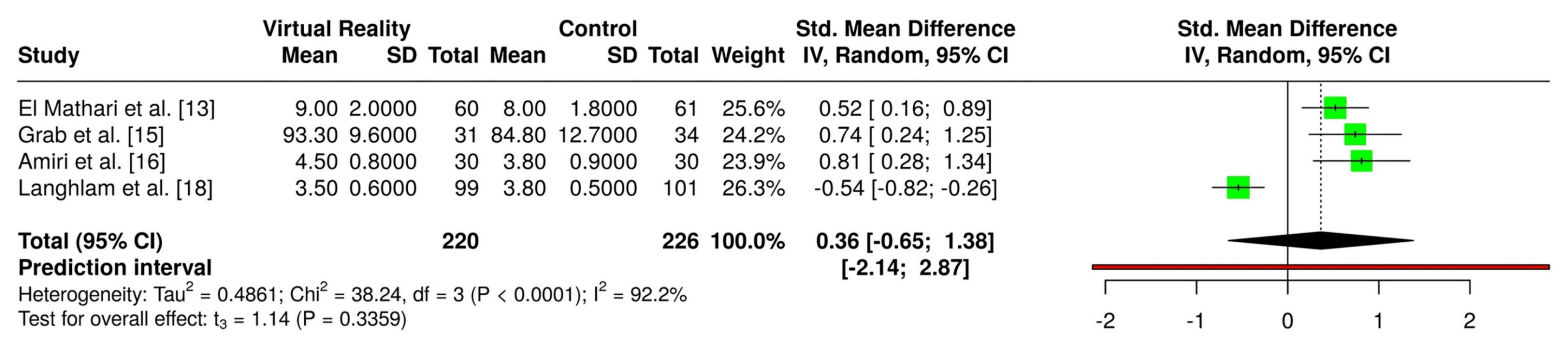
Forest plot comparing VR versus control management for patient satisfaction outcomes in cardiac surgery patients (random-effects meta-analysis) Forest plot displaying pooled estimates comparing VR interventions with standard care for patient satisfaction outcomes in cardiac surgery. While individual studies reported variable results, the overall analysis showed no statistically significant difference between the VR and control groups. Considerable heterogeneity was observed across studies. VR, virtual reality

**Figure 6:**
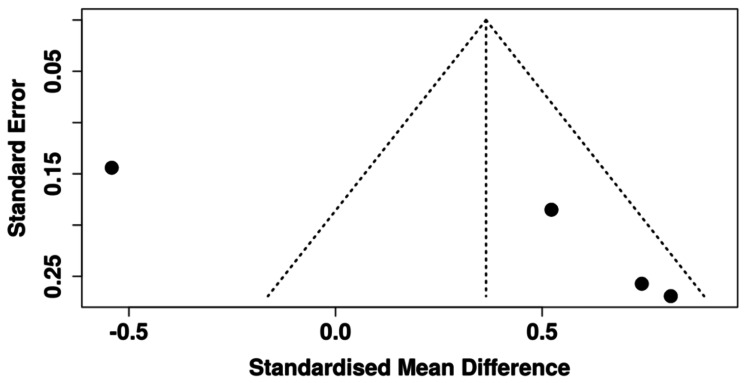
Funnel plot evaluating publication bias for patient satisfaction outcomes in cardiac surgery patients Funnel plot illustrating the relationship between standard error and standardized mean difference for studies assessing patient satisfaction outcomes. The plot demonstrates a roughly symmetrical distribution, suggesting minimal evidence of publication bias [13,15,16,18].

Four RCTs reported post-intervention patient satisfaction outcomes and were included in the quantitative synthesis [13,15,16,18]. Together, these trials comprised 446 patients (220 VR, 226 control). A random-effects meta-analysis demonstrated a nonsignificant trend toward higher satisfaction with VR compared to controls (SMD = 0.36, 95% CI -0.65 to 1.38; p = 0.34). Substantial heterogeneity was observed (I² = 92.2%), indicating inconsistency in effect size and direction across studies. Funnel plot analysis and Egger’s test (p = 0.11) did not suggest publication bias. These results are visualized in the forest plot in Figure 5 and the funnel plot in Figure 6.

Results varied between trials. Both El Mathari et al. and Grab et al. demonstrated significantly greater satisfaction in the VR group compared with conventional education methods [13,15]. Amiri et al. similarly found VR to be associated with higher satisfaction compared with standard 2D video education [16]. In contrast, Laghlam et al. reported greater satisfaction with Kalinox (standard care) compared to VR during chest drain removal post cardiac surgery [18].

Effect of VR on Postoperative Pain in Cardiac Surgery Patients

Postoperative pain outcomes were reported in only two of the included trials [18,19]. Laghlam et al. compared immersive VR with Kalinox during chest drain removal in 200 post-cardiac surgery patients and found that pain intensity, measured by the NRS, was significantly higher in the VR group immediately after the procedure (median 5.0 (IQR 3-7) versus 3.0 (2-6), p = 0.009). No difference was observed 10 minutes after drain removal [18]. Rousseaux et al. assessed pain using a VAS across control, hypnosis, VR, and combined VR-hypnosis groups and reported an overall postoperative increase in pain across all groups, with no significant between-group differences [19]. With only two trials available and differing comparators and contexts, a quantitative synthesis was not feasible for postoperative pain assessment.

Effect of VR on Physiological Outcomes in Cardiac Surgery Patients

Physiological outcomes were variably reported across trials, with heterogeneous measures ranging from standard vital signs to advanced neurophysiological and biomarker assessments.

Amiri et al. demonstrated that VR significantly reduced heart rate, blood pressure, and respiratory rate compared with a standard video intervention, suggesting modulation of autonomic arousal [16]. In contrast, Subramaniam et al. found that both VR and tablet-based interventions produced small reductions in heart rate, but no between-group differences were observed [14]. Similarly, Rousseaux et al. reported no significant effects of VR, hypnosis, or their combination on heart rate, arterial pressure, respiratory rate, or oxygen saturation in the perioperative setting [19]. Laghlam et al. assessed autonomic responses using the Analgesia/Nociception Index and found no difference between VR and Kalinox during chest drain removal [18].

Beyond conventional physiological measures, Tarasova et al. demonstrated that VR multitask training modulated postoperative EEG activity by increasing alpha power and preventing the rise in theta activity typically associated with cognitive dysfunction, indicating a potential neuroprotective effect [17]. Hendricks et al. reported reductions in subjective tension, strain, and upset following VR, which were interpreted as markers of reduced physiological stress, although objective vitals were not measured [21]. Finally, Sadlonova et al. found no significant differences in postoperative cytokine responses (IL-6 and IL-8) but noted a trend toward lower IL-6 increases and significantly shorter hospital stays in the multimodal intervention group, which included VR [20].

Other Outcomes Reported in the Use of VR in Cardiac Surgery Patients

Opioid consumption was infrequently reported across the studies included in this review. Laghlam et al. observed very low peri-procedural morphine use during chest drain removal, with no significant difference between VR and Kalinox [18]. Similarly, Rousseaux et al. reported that on-demand opioid use in the immediate postoperative period did not differ across control, hypnosis, VR, or VR-hypnosis groups [19].

Adverse effects of VR were generally mild, rare, and self-limiting. Laghlam et al. noted transient vertigo and nausea in a small number of patients, while Subramaniam et al. reported occasional eye strain [14,18]. Grab et al. documented that some patients were excluded prior to randomization due to dizziness or difficulty with VR equipment, but no adverse effects were seen in those who proceeded with the intervention [15]. Rousseaux et al. reported no VR-related adverse events [19]. Other trials, including Amiri et al., El Mathari et al., Hendricks et al., and Sadlonova et al., did not report any adverse effects [13,16,20,21]. Across all studies, no serious VR-related complications were described [13-21].

Beyond opioid use and adverse events, several studies reported additional exploratory outcomes. Sadlonova et al. found that VR-containing interventions were associated with shorter hospital stays and improved postoperative self-efficacy compared with standard care [20]. Similarly, El Mathari et al. reported greater perceived preparedness for surgery in patients who received VR education [13]. Feasibility and acceptability were also consistently supported, with Subramaniam et al. noting no withdrawals due to VR and Grab et al. reporting only minor pre-randomization exclusions for dizziness or equipment handling issues [14,15].

Discussion

This systematic review and meta-analysis evaluated the role of VR as a perioperative adjunct in cardiac surgery, synthesizing evidence across nine RCTs [13-21]. VR interventions were consistently associated with higher patient satisfaction, while findings for preoperative anxiety and postoperative pain were mixed and did not demonstrate significant pooled effects. Physiological outcomes showed heterogeneous results, with some trials indicating modulation of autonomic or neurophysiological responses, though overall evidence was inconclusive. Importantly, VR was well tolerated across all studies, with adverse events limited to minor, self-limiting symptoms and no serious complications reported. Collectively, these findings highlight VR’s potential as a feasible and acceptable nonpharmacological adjunct in cardiac surgical care, while underscoring the need for larger, standardized trials to clarify its efficacy across clinical endpoints.

Across seven RCTs including 670 participants, our meta-analysis demonstrated a nonsignificant trend toward reduced preoperative anxiety with VR compared to controls (SMD = -0.23, 95% CI -0.50 to 0.04, p = 0.079) [13-16,18,19,21]. Heterogeneity was low (I² = 37.4%), suggesting that effect sizes were relatively consistent despite differences in intervention design. Funnel plot inspection and Egger’s test (p = 0.154) did not indicate publication bias. Although the pooled effect did not reach statistical significance, the direction of benefit is clinically meaningful in the context of cardiac surgery, where heightened anticipatory anxiety has been linked to poorer recovery, longer hospitalization, and increased postoperative complications [22]. Several trials, including Amiri et al. and Hendricks et al., reported significant anxiety reductions, while others showed neutral effects, particularly when VR was compared with active controls such as tablet-based education or inhaled analgesia [13-16,18,19,21]. These discrepancies likely reflect heterogeneity in VR content, intervention timing, and comparator groups.

Emerging mechanistic evidence may help explain VR’s potential anxiolytic effects. Park et al. demonstrated that VR produced greater anxiety reduction in highly stressful awake surgical procedures, proposing that VR operates not only via attentional distraction but also through cortical modulation of the amygdala, analogous to the gate control theory of pain [23]. This suggests that VR could be particularly effective in populations experiencing intense anticipatory anxiety, such as those awaiting major cardiac surgery.

Our findings parallel broader surgical literature, where VR has consistently reduced perioperative anxiety in orthopedic and pediatric surgery and in interventional cardiology [8,10,11]. The lack of clear significance in cardiac surgery may reflect the unique psychological burden of these operations, which may attenuate relative effect sizes. Nevertheless, the consistency of direction, coupled with promising mechanistic data, supports VR’s potential as a low-risk adjunct in this setting.

The meta-analysis demonstrated a nonsignificant trend toward higher patient satisfaction with VR compared to control conditions (SMD = 0.36, 95% CI -0.65 to 1.38, p = 0.34), with substantial heterogeneity (I² = 92.2%). Funnel plot inspection and Egger’s test (p = 0.11) did not indicate publication bias.

Although statistical significance was not achieved, patients consistently reported positive experiences with VR, suggesting strong acceptability and engagement during cardiac surgery. This may indicate that VR has a more beneficial role in enhancing patient satisfaction through immersive preoperative education rather than replacing conventional comfort measures during procedures. These findings are consistent with broader perioperative research. Caruso et al. demonstrated that pediatric patients experienced high satisfaction with VR during vascular access procedures despite neutral pain outcomes, reflecting strong acceptability of the intervention [24]. Similarly, Haisley et al. found that most patients in the VR group reported a positive experience and willingness to use VR again after foregut surgery, even when satisfaction scores did not differ from controls [25]. In head and neck surgery, Pandrangi et al. reported high satisfaction in both VR and control groups, indicating that VR achieves at least parity with standard care while offering novel experiential benefits [26].

The evidence evaluating the effect of VR on postoperative pain in cardiac surgery remains limited, with only two RCTs addressing this outcome [18,19]. Laghlam et al. reported that pain intensity immediately following chest drain removal was significantly higher in the VR group compared with patients receiving Kalinox, though this difference resolved within 10 minutes [18]. Similarly, Rousseaux et al. found no significant differences in postoperative pain between VR, hypnosis, VR-hypnosis, and standard care groups, despite an overall increase in pain across all interventions [19]. Collectively, these findings suggest that VR has not yet demonstrated consistent analgesic benefit in the postoperative cardiac setting.

The heterogeneity in observed effects may be explained by the mechanisms through which VR modulates pain perception. By diverting attentional focus and providing immersive multisensory input, VR can attenuate the salience of nociceptive stimuli and influence cortical regions involved in pain processing, such as the anterior cingulate cortex and insula [27]. This mechanism appears effective in procedures that are shorter and less invasive, as demonstrated in prior meta-analyses of percutaneous interventions and ablation procedures [11]. However, in cardiac surgery, where postoperative pain is intense, diffuse, and sustained, brief VR interventions may provide insufficient exposure to produce measurable analgesic effects. Despite these limitations, given its favorable safety profile and potential to reduce reliance on pharmacologic analgesia, VR remains a promising nonpharmacologic adjunct, warranting larger, methodologically rigorous trials to clarify its role in cardiac surgical pain management.

Patterns of physiological response to VR during cardiac surgery remain heterogeneous and context dependent. Amiri et al. reported significant reductions in heart rate, blood pressure, and respiratory rate following VR exposure compared with standard video, suggesting attenuation of autonomic arousal [16]. In contrast, Subramaniam et al. and Rousseaux et al. found minimal or no between-group differences in standard vital parameters, while Laghlam et al. observed comparable responses between VR and Kalinox when assessed using the Analgesia/Nociception Index [14,18,19]. More exploratory measures revealed additional variability: Tarasova et al. documented EEG patterns indicative of reduced postoperative cognitive strain, whereas Sadlonova et al. detected no significant changes in inflammatory cytokine activity [17,20]. Such inconsistency likely stems from variation in intervention timing, comparator selection, and the sensitivity of physiological endpoints.

Nonetheless, the capacity of VR to modulate cardiovascular and neurophysiological responses in some studies supports its potential to influence autonomic regulation and perioperative stability. Comparable findings have been observed beyond cardiac surgery. Moharam et al. demonstrated significant reductions in perioperative heart rate and blood pressure during total hip arthroplasty, underscoring VR’s ability to favorably alter autonomic tone during invasive procedures [8].

Collectively, these findings suggest that VR may mitigate stress-related physiological responses by engaging immersive distraction and activating parasympathetic pathways that counteract sympathetic drive. In cardiac surgery, where autonomic imbalance contributes to complications such as arrhythmias, hemodynamic lability, and delayed recovery, this represents a promising avenue for perioperative optimization.

Beyond anxiety, pain, satisfaction, and physiological markers, several studies also explored secondary outcomes such as opioid use, safety, and feasibility. Across the included trials, VR did not significantly reduce perioperative opioid consumption. Both Laghlam et al. and Rousseaux et al. reported comparable morphine use between VR and control groups, suggesting that any analgesic benefit of VR may not consistently translate into reduced pharmacologic demand in cardiac surgery [18,19]. By contrast, opioid-sparing effects have been demonstrated in other surgical contexts, such as head and neck procedures, highlighting the influence of surgical setting, intervention timing, and baseline analgesic practices on observed outcomes [26].

VR was consistently well tolerated, with adverse effects rare, mild, and self-limiting, typically transient dizziness, nausea, or eye strain, with no serious complications reported [14,15,18]. This favorable safety profile stresses the feasibility of integrating VR into routine perioperative care. Several studies also reported additional benefits. Sadlonova et al. observed shorter hospital stays and improved postoperative self-efficacy with multimodal interventions incorporating VR, while El Mathari et al. found enhanced preparedness for surgery following VR education [13,20]. Similarly, Tarasova et al. demonstrated neurocognitive benefits at the cortical and limbic levels, with increased alpha-band activation in the parahippocampal and posterior cingulate regions (Brodmann Area 30) and modulation of frontal cortical activity (middle and superior frontal gyri), indicating engagement of neural networks related to spatial memory, attention, and cognitive control [17].

Taken together, although the direct impact of VR on opioid use remains inconclusive, the intervention appears safe, feasible, and potentially beneficial across domains extending beyond pain control. These findings support VR’s role as a patient-centered adjunct that may enhance psychological resilience, recovery confidence, and overall perioperative experience.

Limitations

Several limitations should be acknowledged when interpreting the findings of this review. The available RCTs investigating VR in cardiac surgery remain few and are predominantly single-center studies with modest sample sizes, which limits statistical power and generalizability. Considerable heterogeneity was also evident across interventions, with variation in VR content, duration, timing, and delivery platforms likely contributing to inconsistent findings across studies. Methodological concerns were frequent, as many trials exhibited some risk of bias arising from incomplete blinding, selective outcome reporting, or the predominance of subjective endpoints such as anxiety and pain.

Outcome measures lacked consistency. While anxiety and satisfaction were commonly assessed using validated scales, pain and physiological outcomes were measured with variable tools, and few studies examined longer-term or objective parameters such as functional recovery, neurocognitive outcomes, or quality of life.

The potential for publication bias must also be considered, given the small evidence base and potential underreporting of neutral or negative findings. These factors collectively emphasize the need for larger, multicenter randomized trials employing standardized VR protocols, rigorous methodology, and consistent outcome reporting to better define the role of immersive technologies in perioperative cardiac surgical care.

The overall certainty of evidence across outcomes was assessed using the GRADE approach, which indicated moderate confidence for anxiety and satisfaction outcomes but low to very low confidence for pain, physiological, and opioid-related measures, as demonstrated in Table 2. These findings reflect the small number of trials, heterogeneity in interventions and measurement tools, and limited blinding feasibility inherent to VR-based studies.

**Table 2:** GRADE certainty of evidence assessment for included outcomes Certainty of evidence for preoperative anxiety and patient satisfaction was rated as moderate certainty, supported by consistent trends favoring VR and low publication bias. Postoperative pain, physiological outcomes, and opioid use were downgraded to low certainty due to heterogeneity, small sample sizes, and imprecision. Safety outcomes were rated as high certainty, with consistent findings demonstrating VR to be well tolerated and free of serious adverse effects. GRADE, Grading of Recommendations Assessment, Development and Evaluation; RCT, randomized controlled trial; VR, virtual reality

Outcome	Number of studies	Study design	Risk of bias	Inconsistency	Indirectness	Imprecision	Publication bias	Overall certainty	References
Preoperative anxiety	Seven studies (meta-analysis)	RCTs	Some concerns (blinding and subjective outcomes)	Low (I² = 37.4%)	No serious indirectness	Some imprecision (95% CI crosses null)	Low risk (symmetrical funnel plot, p = 0.15)	Moderate	[13-16,18,19,21]
Patient satisfaction	Four studies (meta-analysis)	RCTs	Some concerns (allocation concealment and subjective measures)	Substantial (I² = 92.2%)	No serious indirectness	No serious imprecision	Low risk (Egger’s p = 0.11)	Low to moderate	[13,15,16,18]
Postoperative pain	Two studies (narrative synthesis)	RCTs	Some concerns	Serious inconsistency (directionally mixed results)	Some indirectness (different comparators and timing)	Serious imprecision (small samples)	Not assessable	Low	[18,19]
Physiological outcomes	Seven studies (narrative synthesis)	RCTs	Some concerns (measurement bias and variable methods)	Serious inconsistency	Serious indirectness (heterogeneous markers and timings)	Serious imprecision	Not assessable	Low	[14,16-19,20,21]
Opioid use	Two studies (narrative synthesis)	RCTs	Some concerns	Not applicable (limited data)	Some indirectness (nonstandardized reporting)	Serious imprecision	Not assessable	Low	[18,19]
Safety/adverse events	Nine studies (narrative synthesis)	RCTs	Low risk	No inconsistency	No indirectness	No imprecision	Low risk	High	[13-21]

## Conclusions

This systematic review and meta-analysis provide the first comprehensive synthesis of randomized evidence evaluating VR in cardiac surgery. VR was found to be a safe and well-tolerated adjunct, with trends suggesting modest improvements in patient satisfaction and reductions in anxiety, though these did not reach statistical significance. Findings for postoperative pain, opioid use, and physiological outcomes were inconsistent, indicating that VR may not confer clear advantages over standard perioperative care. Nevertheless, its strong feasibility, minimal adverse effects, and potential to enhance patient engagement highlight its clinical promise.

Future research should prioritize larger, multicenter randomized trials employing standardized VR protocols, consistent outcome measures, and long-term follow-up to determine its true therapeutic value. Furthermore, expanding on underlying autonomic and neurocognitive mechanisms may further clarify how immersive technologies can optimize recovery and psychological well-being in cardiac surgical populations.
